# Mice lacking the transcriptional regulator Bhlhe40 have enhanced neuronal excitability and impaired synaptic plasticity in the hippocampus

**DOI:** 10.1371/journal.pone.0196223

**Published:** 2018-05-01

**Authors:** Kelly A. Hamilton, Yue Wang, Sophia M. Raefsky, Sean Berkowitz, Ryan Spangler, Caitlin N. Suire, Simonetta Camandola, Robert H. Lipsky, Mark P. Mattson

**Affiliations:** 1 Laboratory of Neurosciences, National Institute on Aging, Baltimore, Maryland, United States of America; 2 Krasnow Institute for Advanced Study, George Mason University, Fairfax, Virginia, United States of America; 3 Department of Neurosciences, Inova Health System, Falls Church, Virginia, United States of America; Nathan S Kline Institute, UNITED STATES

## Abstract

Bhlhe40 is a transcription factor that is highly expressed in the hippocampus; however, its role in neuronal function is not well understood. Here, we used *Bhlhe40* null mice on a congenic C57Bl6/J background (*Bhlhe40* KO) to investigate the impact of Bhlhe40 on neuronal excitability and synaptic plasticity in the hippocampus. *Bhlhe40* KO CA1 neurons had increased miniature excitatory post-synaptic current amplitude and decreased inhibitory post-synaptic current amplitude, indicating CA1 neuronal hyperexcitability. Increased CA1 neuronal excitability was not associated with increased seizure severity as *Bhlhe40* KO relative to +/+ (WT) control mice injected with the convulsant kainic acid. However, significant reductions in long term potentiation and long term depression at CA1 synapses were observed in *Bhlhe40* KO mice, indicating impaired hippocampal synaptic plasticity. Behavioral testing for spatial learning and memory on the Morris Water Maze (MWM) revealed that while *Bhlhe40* KO mice performed similarly to WT controls initially, when the hidden platform was moved to the opposite quadrant *Bhlhe40* KO mice showed impairments in relearning, consistent with decreased hippocampal synaptic plasticity. To investigate possible mechanisms for increased neuronal excitability and decreased synaptic plasticity, a whole genome mRNA expression profile of *Bhlhe40* KO hippocampus was performed followed by a chromatin immunoprecipitation sequencing (ChIP-Seq) screen of the validated candidate genes for Bhlhe40 protein-DNA interactions consistent with transcriptional regulation. Of the validated genes identified from mRNA expression analysis, insulin degrading enzyme (*Ide*) had the most significantly altered expression in hippocampus and was significantly downregulated on the RNA and protein levels; although Bhlhe40 did not occupy the *Ide* gene by ChIP-Seq. Together, these findings support a role for Bhlhe40 in regulating neuronal excitability and synaptic plasticity in the hippocampus and that indirect regulation of *Ide* transcription may be involved in these phenotypes.

## Introduction

Basic helix loop helix enhancer 40 (Bhlhe40; also known as Clast5, Stra13, Sharp2, Dec1, Eip1, Cr-8, and Bhlhb2) is a transcription factor that: 1) directly represses gene expression via binding to class B E-Box sequences (CACGTG) [1]; 2) directly activates gene expression by binding to Sp1 sites [2,3]; and 3) indirectly regulates gene expression by interacting with basal transcription machinery, other transcription factors, or histone modifiers [4–6]. *Bhlhe40* is involved in a number of essential functions such as hypoxia, DNA damage responses, and metabolism and is highly expressed in the mouse brain, primarily in hippocampus and cortex; however, its role in hippocampal function is not well understood [4,6–9]. It was previously found that rats injected with the convulsant kainic acid (KA) have increased *Bhlhe40* expression in hippocampus and cortex [9]. In particular, the hippocampus is known to be a frequent focus of seizures and KA injection to the hippocampus is a common model for temporal lobe epilepsy [10]. Together, these findings suggested that Bhlhe40 may be involved in the seizure response.

In a mixed genetic background line of mice deficient for *Bhlhe40* (*Bhlhe40* KO-mix), a single intraperitoneal injection of KA resulted in enhanced seizure severity compared to littermate controls containing the gene [11]. Furthermore, *Bhlhe40* KO-mix mice had enhanced hippocampal brain derived neurotrophic factor *(Bdnf)* gene expression from one of the known activity-dependent promoters of the *Bdnf* gene, promoter 4, suggesting a possible role for altered BDNF signaling in seizure susceptibility [12,13]. The same study found that Bhlhe40 occupancy of an E-box binding site in promoter 4 of the *Bdnf* gene was lost upon N-Methyl-D-Aspartate receptor (NMDAR) activation, suggesting that Bhlhe40 suppresses at least one activity-dependent gene target at baseline and that Bhlhe40 may have a role in neuronal excitability by regulating activity-dependent *Bdnf* gene expression [11].

Here, using a line of *Bhlhe40* null mice on a pure C57Bl/6 background, we sought to test whether Bhlhe40 is involved in neuronal excitability, as well as synaptic plasticity, by direct measurement in hippocampal slices of *Bhlhe40* KO animals followed by investigation of behavioral correlates, i.e. seizure susceptibility, and learning and memory, respectively. This study directly measured the role of *Bhlhe40* in two major functions of the hippocampus: neuronal excitability and synaptic plasticity. Further, we performed a whole genome expression array and validation studies to discover genes that Bhlhe40 may regulate, in addition to *Bdnf*.

## Materials and methods

### Mice

*Bhlhe40* KO mice were generated as described previously [11] and backcrossed for six generations into the C57Bl/6 background strain to generate the congenic mouse model. Animals in this study were housed at the National Institute on Aging facility on a 12 hour light/dark cycle (6:30am-6:30pm) and allowed free access to food and water. Experiments used only male mice with the exception of a female mouse used for validation of the chromatin immunoprecipitation assay. This research was approved by the National Institute on Aging Animal Care and Use Committee and was performed according to guidelines in the NIH Guide for the Care and Use of Laboratory Animals.

### Electrophysiology

Hippocampal slices were prepared using procedures described previously [14]. Briefly, transverse slices of whole brain were cut at a thickness of 350 μm, and were allowed to recover for 1–6 hours in a holding chamber in artificial cerebral spinal fluid (ACSF), bubbled with 95/5% (O_2_/CO_2_) at room temperature. Slices were from 3–4 month old mice for field recordings and P12-21 pups for whole-cell recordings. Field potentials were recorded from CA1 stratum radiatum using glass electrodes (1–3 MΩ) filled with oxygenated ACSF. Schaffer collaterals were stimulated using bipolar tungsten electrodes at 0.033Hz and 30 μs duration. The stimulation intensity was set to 30–40% of the maximum EPSP amplitude. LTP was induced by high frequency stimulation (100 Hz, 1s) and LTD was induced by low frequency stimulation (1 Hz, 15 min). The ACSF for field recordings contained 50 μM picrotoxin to block GABA(A) activity. Slices were pre-selected for those with a steep input-output curve. During recording, slices were warmed to 30–32°C. Whole-cell excitatory postsynaptic currents (EPSCs) and inhibitory postsynaptic currents (IPSCs) were recorded from CA1 pyramidal neurons [15]. The neurons were visualized by differential interference contrast microscopy using a 40X water immersion lens. Series resistance was 6 to 10 MΩ. The patch electrode (3–5 MΩ) contained (in mM): 125 CsMeSO_3;_ 2.5 CsCl; 0.2 EGTA, 7.7 TEA; 20 HEPES; 8 NaCl, 4 Mg-ATP; 0.3 Na-GTP; 5 QX-314; pH 7.2; the osmolality was 280–290 mmol/kg. Data were collected using an Axopatch 200B and MultiClamp 700B (Molecular Devices, Sunnyvale, CA) amplifier. Signals were filtered at 2 kHz, digitized at 10 kHz, and analyzed using pCLAMP 8 software (Molecular Devices). Data was presented as the mean ± SEM and statistical comparisons were made using unpaired t-tests and Mann-Whitney Rank Sum Test.

### Seizure model and analyses

Six WT and six *Bhlhe40* KO 5 month old mice were anesthetized with isoflurane and implanted with a cannula (23XX gauge cannula with a 26TW gauge stylus; Vita Needle) to dorsal hippocampus (from Bregma: 1.8 mm posterior, 1.5 mm lateral, 2.0 mm advanced ventrally from the dura). Mice were allowed two weeks to recover, then infused with 200 nL of saline containing 227 ng of kainic acid (KA) (Abcam; Cat#: Ab120100; Batch Molecular Weight: 226.74;) in sterile 0.9% saline [16]. Animals were video monitored for six hours for behavioral seizure analysis according to a seizure scale described previously [17]: stage 1 freezing behavior; stage 2, rigid posture and/or tail extension; stage 3, myoclonic jerks of head and brief twitching; stage 4, forelimb clonus and partial rearing; stage 5, forelimb clonus with rearing and falling; stage 6, tonic-clonic seizures. Additionally, stage 7 was denoted for one animal that died. To avoid falsely labeling normal pausing behavior as seizure activity, only scores of 2 or greater were considered as seizure activity. All mice were infused at the same time of day (12 pm) with one *Bhlhe40* KO and one WT infused each day for six consecutive days, with the infusion order counterbalanced.

For histological analyses, mice were euthanized three days after KA infusion and were perfused transcardially with formalin and the brain removed. Following overnight fixation, the brains were washed with PBS and transferred to 30% sucrose solution for at least two days before sectioning at 40 μm thickness on a sliding microtome. Sections were stained with 1% cresyl violet acetate solution to assess cell death. Specifically, cell death was assessed on cresyl violet stained sections at 10X magnification by measuring the intensity of the CA3 cell body layer relative to stratum radiatum intensity for background, and by measuring the thickness of the CA3 cell body layer at the genu to measure for cell loss and apoptotic swelling, respectively, using Fiji software. Cell death in area CA3 was quantified due to the high vulnerability of CA3 neurons to KA excitotoxicity [18].

A separate set of adult 3–4 month old mice were implanted with cannulas (described above) together with a subcutaneous radio transmitter (Data Sciences International; F20-EET) connected to a brain surface electrode above the primary motor cortex (from Bregma: 0.6 mm anterior, 1.7 mm lateral) [19,20] in the hemisphere contralateral to that in which the cannula was implanted. Mice were given three weeks to recover, and for the first five days were given twice daily injections of an antibiotic (Baytril) and a nonsteroidal anti-inflammatory drug (Carprofen), to prevent infection and discomfort. EEGs were recorded for 72 hours of baseline, 72 hours after infusion of 0.9% NaCl saline (solvent for KA), and 72 hours after infusion of a low dose of KA (200 nL of 57 ng) to prevent any possible animal death and to prevent saturation of the EEG signal from high activity. Power analysis was performed on EEG signals using Neuroscore software (Data Sciences International). Power bands for delta (1–4 Hz) and Theta/Alpha (6–10 Hz) were used as surrogate signals for behavioral seizures scores of 1–3 (freezing through forelimb clonus) and 4–6 (seizures with rearing/falling), respectively [21]. Additionally, Gamma (30–50 Hz) power was assessed as it is seen immediately after KA infusion and prior to behavioral evidence of seizures [10].

### Morris water maze testing

12 WT and 13 *Bhlhe40* KO mice (4–7 month old) were trained for six days to find the hidden platform of the Morris water maze (MWM). The MWM used was a circular tank 140 cm in diameter with a 17cm diameter platform. The MWM was divided into four quadrants (North-West, North-East (NE), South-East, South-West (SW)) with a designated thigmotaxis outer ring 17cm from the wall of the maze. The water temperature was set to 24°C and non-toxic white paint was added to the water to obscure the submerged platform. Visual cues were on the walls for the mice to use to spatially orient to the platform. The platform was placed in the NE quadrant for initial training. The training consisted of a 60 second trials at each of the four start locations (North, South, East, and West) in a randomized order each day. If the animal was unable to find the platform in 60 seconds during the training, the experimenter guided the animal to the platform and allowed the animal to stay on the platform for 15 seconds. Recollection of the platform location was assessed on probe trials performed at 4-, 24-, and 48-hours post training. Immediately following the 48-hour probe, a test for visual impairments and motivation was conducted by returning the platform to the pool in a different location than the animals were trained on and affixing a small blue flag to the platform to allow animals to easily see the platform and to escape. Seven days later (14 days after the start of MWM training), the above procedure was repeated, training the animals to the opposite platform location (SW quadrant; MWM reversal task). No visual task was performed at the end of the reversal experiment. Video tracking software (ANY-maze) was used to track and analyze the animals’ performance on the MWM.

Statistical Analysis was performed using GraphPad PRISM statistical software. Training trials were analyzed for latency to find the platform across days by two-way repeated measures (RM)-ANOVA (Genotype x Training Day) and linear regression to compare slope and elevations of the regression lines. Probe trials were analyzed for time spent in each zone (each of the four quadrants, including the platform area), number of entries into the platform area, and latency to platform by two-way RM-ANOVA (Genotype x Probe Trial). MWM probes were also analyzed for the latency to platform zone entry, and latency to initial goal quadrant on the reversal probes, by linear regression to compare slope and elevations of the regression lines across the 4-, 24-, and 48hr probes between genotypes. The Mann Whitney U test was used to test for individual differences on the latency to platform area and percent time in quadrant (SW vs NE) on the reversal probes and to test for a difference in swim speed. One *Bhlhe40* KO animal was found to exhibit continued thigmotaxis behavior across all conditions and was not included in the analysis (*Bhlhe40* KO n = 12 for analysis). A second *Bhlhe40* KO animal was not included in the initial 24hr probe analysis due to a software recording error (*Bhlhe40* KO n = 11 for that condition).

### Gene expression

#### Brain extraction and fresh tissue dissection

Animals were fasted overnight prior to isoflurane anesthesia, decapitation, and brain removal. On ice, the hippocampus was isolated from the brain as described previously [22]. Cortex and cerebellum were then dissected. Isolated tissue was snap frozen on dry ice then stored at -80°C until later use.

#### Gene expression array

Hippocampus, cortex, and cerebellum were isolated from naïve 3–5 month old WT (n = 4) and *Bhlhe40* KO (n = 4) mice between 10:30 am and 1 pm. RNA was extracted from brain tissue samples using an electronic tissue homogenizer in 1 mL Qiazol, followed by spin column isolation according to the manufacturer’s protocol (Qiagen; Cat# 74104). RNA was treated with RNase-free DNase (Qiagen; Cat# 79254) during RNA isolation. The quality of the RNA was verified using an Agilent Bioanalyzer RNA 6000 Chip (Agilent, Santa Clara, CA). 500 ng of RNA was then amplified to create biotinylated antisense RNA using an Illumina^®^ TotalPrepTM RNA amplification kit according to the manufacturer’s instructions. Biotinylated antisense RNA (750 ng) was hybridized overnight to mouse Ref8 v2 BeadChip microarrays (Illumina, San Diego, CA). Following post-hybridization rinses, arrays were incubated with streptavidin-conjugated Cy3, and scanned at 0.53 microns using an Illumina iScan scanner. Hybridization intensity data were extracted from the scanned images, and evaluated using Illumina GenomeStudio software, V2011.1.

#### Gene expression array significance determination

Raw microarray florescent signal data were filtered by the detection p-value and Z normalization to obtain normalized probe signals. Sample quality was first analyzed by scatter plot, principal component analysis, and gene sample hierarchy clustering to exclude possible outliers. The sample two-way hierarchical clustering was performed on all array probes using normalized Z scores to get sample class information. The sample was assumed to have the classification of the sample group. If the sample deviated far from the group, sample correlation analysis was done to identify the possible outlier. Principal component analysis (PCA) was performed using normalized Z scores of gene expression for all samples and probes. Each sample group was assigned a color for identification. The sample coordinators on the first three most dominant principal component planes were illustrated by a three-dimensional scatter plot with sample group colors and sample names. One-way ANOVA test on the sample groups were used to eliminate the probes/genes with larger variances within each comparing group. ANOVA p-values less than 0.05 were adopted for each probe/gene as a global quality filter. Genes were determined to be differentially expressed after calculating the gene expression change scale by Z-ratio, which indicates the fold-difference between experimental groups, and correcting for multiple comparison error level by false discovery rate (FDR), which controls for the expected proportion of false rejected hypotheses. Individual genes with p values less than 0.05 and a FDR less than 0.3 were considered significantly different. Hierarchy clustering, and principal components analysis (PCA) were performed to identify patterns within groups and probes affected by various experimental factor effects.

#### Gene expression array pathway analysis

The parameterized analysis of gene enrichment (PAGE) algorithm was employed for gene set enrichment analysis by using all of the genes in each sample as input, and the data set supplied by Gene Ontology Institute and MIT Broad Institute pathway gene set. For each relevant comparison, the lists of differentially expressed genes and Z ratios were entered into the PAGE Pathway Analysis software to organize them according to known biological pathways. The Enrichment Z-scores for each functional grouping were calculated based on the chance of mRNA abundance changes predicting these interactions and networks by Z-test. All pathways considered significant had at least three genes in the microarray gene set. Gene Ontology terms were required for each gene to have a p value less than 0.05 and an FDR less than 0.3. More stringent criteria were used for analysis of canonical pathways, requiring a p value less than 0.01 and an FDR less than 0.1. MIAME compliant Gene Expression Array data has been deposited to the NCBI Gene Expression Omnibus: GSE85959.

#### Quantitative Reverse Transcription Polymerase Chain Reaction (qRT-PCR)

Messenger RNA (mRNA) from 3–5 month old WT (n = 8) and *Bhlhe40* KO (n = 8) naïve mice were extracted from hippocampal tissue as describe above. To control for DNA contamination, hippocampal RNA was treated with RNase-free DNase during RNA isolation, the same as RNA processing for the gene expression array. All tissue dissections were performed between 10:30 am and 1 pm. MRNA from these samples was reverse transcribed to cDNA using Superscript III First-Strand Synthesis Supreme (Invitrogen; Cat#: 18080400). Complementary DNA was amplified using Sybr green 2X master mix (Applied Biosystems; Cat#: 4309155) with primers designed using primer blast (NCBI); thermal cycling: 95°C 10min then cycle 95°C 1min, 72°C 1min, 65°C 1min, 40 times; followed by a melt curve from 70.0°C to 90.1°C with a read every 0.2°C. All primers were intron-spanning with the exception of *Bdnf4*, which was only tested on cDNA generated from RNA that was treated with RNase-free DNase. *Bdnf1* and *Bdnf4* primer sequences were selected because they were used in a prior study [11]. Only primer pairs that generated products with a single peak on the melt curve were used in the analysis. The comparative Ct method was used to determine the normalized changes of the target gene relative to a calibrator reference. Significance was determined by unpaired t-tests. Table 1 shows primer sequences of genes tested and an *Hpr*t control.

**Table 1:** Quantitative RT-PCR Primers.

Gene	Forward Primer	Reverse Primer
*Bdnf 1*	AAGCCGAACTTCTCACATGATGA	TGCAACCGAAGTATGAAATAACCATAG
*Bdnf 4*	CTGCCTAGATCAAATGGAGCTTCT	GGAAATTGCATGGCGGAGGTAA
*Bhlhe41*	CCAAAAGGAGCTTGAAGCGAG	ACCGGCGATTTCAGAGAGC
*Cacna2d1*	ATTTGGACGCCCTGGAACTG	GCTCCTTTGGCTGAAGATTTGG
*Cacnb4*	GAGGGCTGTGAGATTGGCTT	GAGGAATGTGCTCCGTCACT
*Camk1d*	CTCGACACCCATGGATTGCT	ACTACAGAGCGTGGAAGGTG
*Chl1*	GTAGAGGTACTTGTGCCGGG	TCACTGTCGCTGTATTCACCA
*Clcn3*	ATCTGGCAGTTGTGCCTAGC	TCACACCACCTAAGCACGC
*Clock*	CCACAAGGCATGTCACAGTTTC	TTGCTGTATCATGTGCTGGC
*Dbp*	CGCGCAGGCTTGACA	CAGGGCGTTTTCCTTCTCCA
*Gabarap*	GTGCCTTCTGATCTTACAGTTGGTC	ACGCTTTCATCACTGTAGGCA
*Gabbr1*	AGTCGCTGTGTCCGAATCTG	TCTGAGTGTGGCGTTCGATT
*Gabrg2*	CATCTCTGAGTGACGGGACC	GGTTTCACTCCGATGTCAGGT
*Glud1*	GTGGTCGATGTACCGTTTGG	ATGGTGCTGGCATAGGTGTC
*Gria1*	GGGAATGTGGAAGCAAGGACT	AAACAGAAACCCTTCATCCGCT
*Gria2*	AGACTACGACGACTCCCTGG	ACTTGAACCTGCTTGAGGGC
*Gria3*	CTCCAAGATCACTTTCTGGGC	TGGCTCTGCGGATTTCATGT
*Ide*	GACAGAGGAGGCGTTCCAAA	CTTCAGGTTGTGGCAAGGGT
*Kcna1*	CTGAGAGGAGAAGGACGGGA	CGATCGATGGACGCTGGC
*Kcnab1*	TGGAGATCATGGAAGCCTACTC	AGAACTTTCTGGCACCCCAT
*Kcnab3*	TATGAGCACGGCGTAAACCT	TAGCTTGATCTCCTCCAGCCT
*Kcnd2*	CATGACAACACTGGGGTATGG	GCATTTGCACTCCCGCTTTT
*Kcnh1*	GACGACTCCTGCAAAGGTTGG	TAGGATGATGTGAGGGGGTGT
*Kcnh2*	GATCCTGCGGGGTGATGTC	CATGTTGGTATCTCGAAGGTTGAA
*Kcnma1*	GGGTGATGATATCCGCCCAG	GTTGGCTGCAATAAACCGCA
*NTrk3*	CGCCAGCATCAACATTACGG	TGCTCCAGTCTCAATTCCCG
*Ppargc1a*	GCTCAAGCCAAACCAACAACT	GGCCCTTTCTTGGTGGAGTG
*Scn1a*	GGTTACTCTGAACTCGGGGC	AGCCCTTAAATGTGGCAACT
*Scn3b*	AATACCCCTGCGAGTCACTG	GGTAGTCAGACGCATTTTCCTG
*Scn8a*	GTGTCGTGTGGCCCATAAAC	GCCGCTCGTAAGGTCAGC
*Snx14*	CCAAATTCAACAGAAGCACACAA	GCAGCAAGGTTTCGAGGAGT
*Stmn1*	CGACATGGCATCTTCTGATATTC	GAGCACCTCCTTCTCATGCT
*Syngr1*	TTCGACCCCTACACCCTGG	AAGCAGAAACCCACGAACCA
*Hprt* (control)	CCTGCTGGATTACATTAAAGCACTG	CCTGAAGTACTCATTATAGTCAAGG

Intron-spanning primers were designed for this study using Primer Blast (NCBI), except for *Bdnf1* and *Bdnf4* sequences, which are identical to those used in the previous study [10].

#### Western blot analysis

Hippocampi from 3–5 month old WT (n = 5) and *Bhlhe4*0 KO mice (n = 5) were homogenized in RIPA buffer supplemented with a protease inhibitor cocktail (Roche; Cat#: 04693124001). 20 μg proteins were separated on a 4–12% Bis-Tris Gel (Thermo; Cat#: NP0335BOX) and transferred onto 0.45 μm nitrocellulose membranes (Thermo; Cat#: 88025). Membranes were blocked in 5% milk (Thermo; Cat#: 37530) for 1 hour then incubated with primary antibodies overnight at 4°C. Following washes and incubation with the HRP-conjugated secondary antibody for 1 hour the immunoreactive bands were detected by chemiluminescence (Pierce; Cat#: 32106). Relative protein levels were determined by densitometry using Fiji (image J analysis). Significance was determined by unpaired t-tests. Levels of GAPDH were used as an internal loading control. The following antibodies were used in this study: rabbit anti-GluR1 (Abcam; Ab109450); rabbit anti-GABA(B)R1 (Abcam; Ab166604); rabbit anti-Kctd12 (Protein Tech Group; 15523-1-AP); rabbit anti-Camk1d (Origene; TA309027); rabbit anti-IDE (Gene Tex; GTX111664); rabbit anti-Scn1a (Abcam; Ab24820); rat anti-Chl1(Millipore; MABN299); rabbit anti-Bhlhe41 (Biorbyt; orb224120); rabbit anti-Clock (Biorbyt; orb235103); goat anti-GAPDH (Santa Cruz; sc-20357). HRP secondary antibodies against the primary antibodies were used (Jackson Immunoresearch).

#### BDNF ELISAs

Hippocampi from six WT and six *Bhlhe40* KO naïve 3–4 month old mice were homogenized in PBS, centrifuged at 1200 x g for 5 min at 4°C, and the tissue pellet was resuspended in 200 μL M-PER buffer (Thermo Scientific cat#: 78501). Samples were analyzed using a BDNF ELISA kit (Promega; Cat# G7611) and a pro-BDNF ELISA kit (Aviscera Bioscience; Cat# SK00752-08) according to manufacturer’s protocols. Values were determined according to a linear standard curve and normalized by protein concentration which was determined by the BCA method (Pierce; Cat# 23227).

#### Insulin ELISA

Hippocampi from seven WT and Seven *Bhlhe40* KO 4–7 month old mice that had been behaviorally tested were homogenized in 100 μL M-PER buffer (Thermo Scientific cat#: 78501) with protease inhibitors (Roche; Cat#: 04693124001), centrifuged at 12,000 x g for 30 min at 4°C, then supernatant was collected [23]. Insulin levels were quantified by ELISA (EMD Millipore; Cat#: EZRMI-13K) according to the manufacturer’s protocol. Values were interpolated from the variable slope standard curve by non-linear regression (4PL) using PRISM software (Graph Pad; Version 5.0) and normalized by protein concentration via BCA (Pierce; Cat#: 23227).

### Bhlhe40 chromatin immunoprecipitation sequencing (ChIP-Seq)

#### Bhlhe40 antibody validation

Tissue fixation and chromatin isolation were carried out according to the Active Motif (Carlsbad, CA) protocol for ChIP. To validate the assay, Bhlhe40 antibody was used in a ChIP reaction with 19.5 μg of chromatin prepared from the cortex of a single female WT mouse (age 2.6 months). Chromatin was mixed with anti-Bhlhe40 antibody (Novus, cat# NB100-1800, Lot# C-1) and the ChIP DNA was processed into a standard Illumina ChIP-Seq library and sequenced. Antibody validation sequencing generated more than 5 million reads. Reads were aligned to the mouse genome (mm10), producing approximately 9.7 million unique alignments. MACS peak finding [24] was used to identify the most significant peaks using a default p value of 1 e-7, with 11,454 peaks identified. The antibody validation of Bhlhe40 ChIP-Seq indicated that the top motif identified by MEME [25] conformed to the CACGTG consensus E-box recognized by Bhlhe40.

#### Bhlhe40 ChIP-Seq

Subsequently, hippocampi from four male mice (age 2.5 months) were pooled for ChIP-Seq analysis. Results from sequencing indicated 18,030,260 normalized tags from a total of 27,546,895 reads, producing 6,038 peaks. Peaks were called using either Model-based Analysis of ChIP-Seq (MACS) or Spatial Clustering for Identification of ChIP-Enriched Regions (SICER) algorithms [26]. The MACS default cutoff p-value was 1e-5 for narrow peaks and 1e-1 for broad peaks, and SICER default cutoff was FDR 1e-10 with gap parameter of 600 bp.

#### Sequence analysis

The 75-nt sequence reads generated by Illumina sequencing (using NextSeq 500) were mapped to the genome using the BWA algorithm with default settings. Alignment information for each read was stored in the BAM format. Only reads that passed Illumina’s purity filter, aligned with no more than two mismatches, and mapped uniquely to the genome were used in subsequent analysis. In addition, unless stated otherwise, duplicate reads (“PCR duplicates”) were removed.

#### Determination of fragment density

Since the 5´-ends of the aligned reads (“tags”) represented the end of ChIP/IP-fragments, the tags were extended *in silico* (using Active Motif software) at their 3´-ends to a length of 150–250 bp, depending on the average fragment length in the size selected library (normally 200 bp). To identify the density of fragments (extended tags) along the genome, the genome was divided into 32-nt bins and the number of fragments in each bin was determined. This information produced a “signal map” (histogram of fragment densities) and was stored in a separate file (bigWig file) for visualizing peaks of protein-DNA interaction.

#### Data normalization

The tag number of the pooled samples was reduced by random sampling in the input sample.

#### Active region analysis

To compare peak metrics between the pooled samples and input, overlapping Intervals were grouped into “Active Regions,” which were defined by the start coordinate of the most upstream Interval and the end coordinate of the most downstream Interval (union of overlapping Intervals; “merged peaks”). In locations where only one sample had an Interval, this Interval was defined as the Active Region. The use of Active Regions was necessary because the locations and lengths of Intervals are rarely exactly the same when comparing different samples.

#### Annotations

After defining the Intervals and Active Regions, their genomic locations along with their proximities to gene annotations and other genomic features were determined and presented in a data file (Results section). In addition, average and peak (i.e. at “summit”) fragment densities within Intervals and Active Regions were compiled.

## Results

### *Bhlhe40* KO CA1 neurons are hyperexcitable

**Fig 1:**
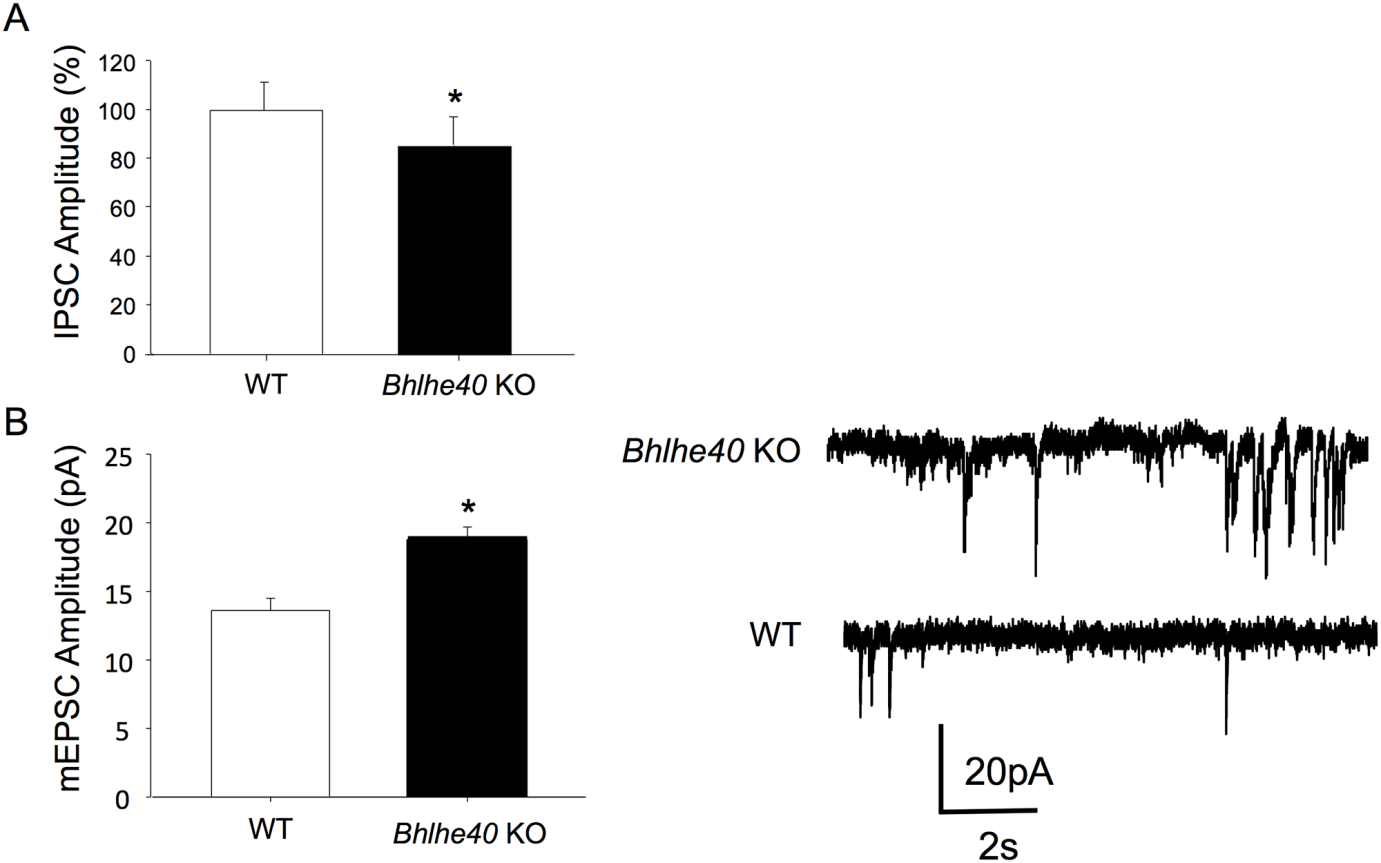
*Bhlhe40* KO mice have increased neuronal excitability. (A) *Bhlhe40* KO mice have a 14.4% decrease in IPSC amplitude (*Bhlhe40* KO: 85.6 ± 12.2, WT: 100 ± 11.6); n = 4 mice for each *Bhlhe40* KO (5 cells) and WT (6 cells). (B) Left: *Bhlhe40* KO mice have a 40% increase in mEPSC amplitude, relative to WT levels (*Bhlhe40* KO: 18.99 ± 0.69 pA, WT: 13.6 ± 0.88 pA). Right: Example traces of mEPSCs from WT and *Bhlhe40* KO slices. Error bars are standard error of the mean; unpaired t-tests were used for both mEPSC and IPSC amplitude comparisons *Bhlhe40* KO hippocampal slices compared to WT slices; * = p<0.05.

In single cell recordings in hippocampal slices we found that CA1 neurons from *Bhlhe40* KO mice had significantly reduced evoked IPSC amplitude in CA1 hippocampal neurons (Fig 1A; 14.4% decrease; p<0.05). Additionally, mEPSC amplitude was significantly greater (Fig 1B; 40% increase; 5.4pA; p<0.01) than WT controls. However, there was no difference in mEPSC frequency (S1 Fig). This suggests that the rate of spontaneous neurotransmitter release is not affected, but the post-synaptic response to a given quantal release is greater. This shows that *Bhlhe40* KO CA1 neurons have a greater excitatory response and a weaker inhibitory response, indicating an overall increase in neuronal excitability.

**Fig 2:**
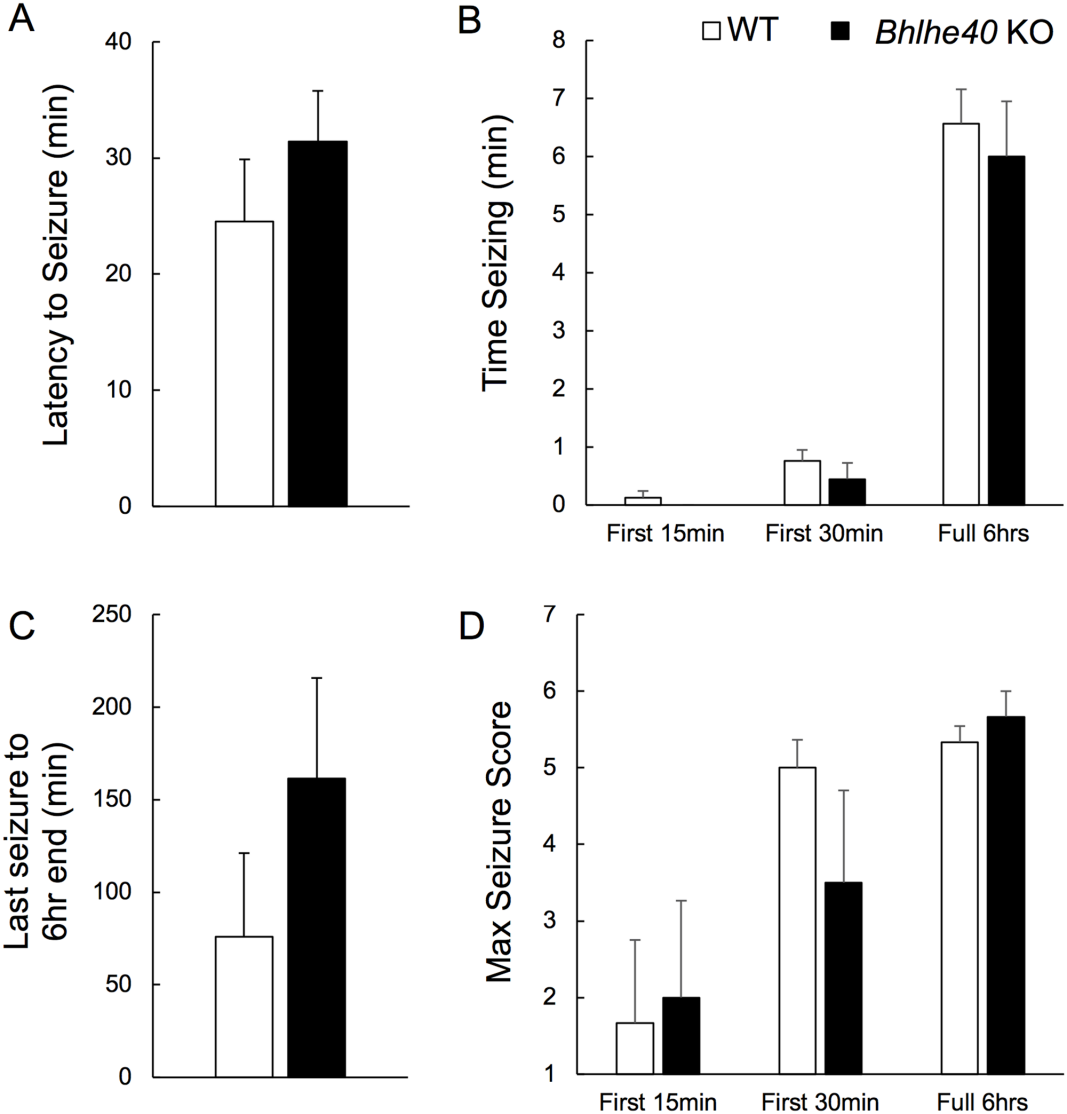
*Bhlhe40* KO mice do not have more-severe behavioral seizures. Animals were infused with 227ngs of KA and monitored for six hours. (A) Latency from infusion to first seizure, unpaired t-test p>0.05. (B) Total time seizing over the first 15 minutes, first 30 minutes, and full 6 hours, unpaired t-test p>0.05. (C) Latency from last seizure to the end of the six-hour monitoring period, unpaired t-test for each p>0.05. (D) Seizure scale for maximum seizure response for the first 15 minutes, first 30 minutes, and full 6 hours, unpaired t-test for each p>0.05. n = 6 for WT mice and n = 5 for *Bhlhe40* KO mice (Figs 2B and 2C), n = 6 for *Bhlhe40* KO mice (Figs 2A and 2D; including an animal that died during the monitoring period). Error bars are standard error of the mean.

Given that hyperexcitability is a feature of epileptiform activity [27] and that *Bhlhe40* KO-mix mice had enhanced seizure responses to KA injection [11], we next investigated whether the same held true for congenic *Bhlhe40* KO mice. Here, we induced behavioral seizures via intra-hippocampal KA infusion in six WT and six *Bhlhe40* KO 5-month old mice. Animals were monitored for six hours following an infusion of 227 ng KA to dorsal hippocampus and assessed according to a seizure scale. There were no significant differences in latency to seizure initiation (Fig 2A), time seizing (Fig 2B), time from last seizure to end of the 6-hour monitoring period (Fig 2C), or maximum seizure score (Fig 2D). Post mortem analysis of the hippocampal region showed comparable levels of cell loss in the CA3 stratum pyramidale neuronal layer relative to CA3 stratum radiatum (S2A and S2B Fig). WT and *Bhlhe40* KO mice exhibited similar electrophysiological manifestations of seizure activity in EEG recordings in animals administered a lower dose of KA (57 ng; S3A, S3B, S3C and S3D Fig).

### *Bhlhe40* KO deficiency results in impaired LTP and LTD at CA1 synapses

**Fig 3:**
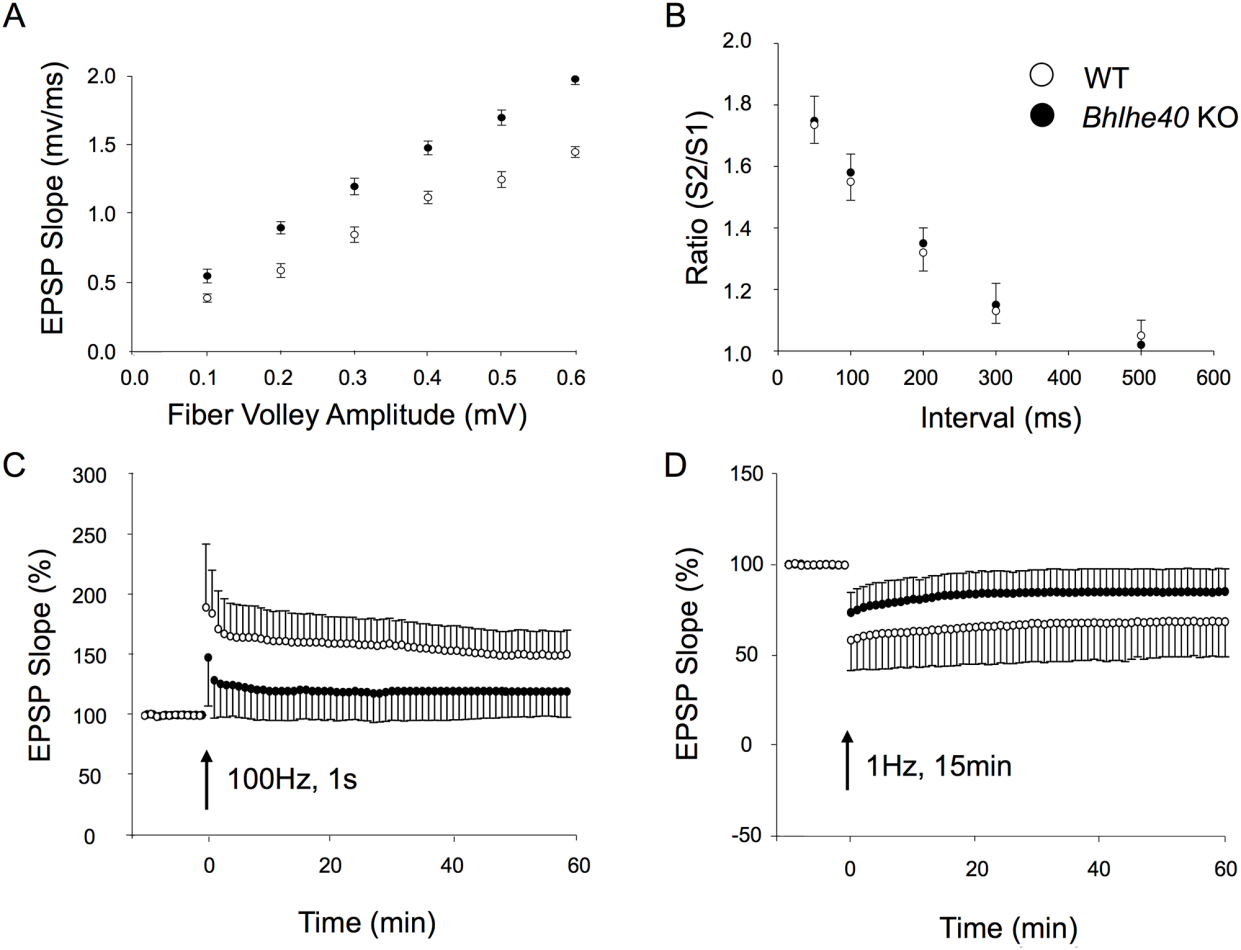
*Bhlhe40* KO mice have reduced hippocampal synaptic plasticity. (A) EPSP slope input/output curves were significantly different (p<0.001; n = 9). (B) Paired pulse facilitation is not significantly impaired (p = 0.063; n = 5). (C) There was a 60% reduction in LTP measured by EPSP slope 1hr following 1s of 100Hz stimulation (*Bhlhe40* KO: 120 ± 20.7%, WT: 150.5 ± 21%; p<0.001; n = 5 mice, 6 slices each). (D) There was a 52% reduction in LTD measured by EPSP slope 1hr following 15min of 1Hz stimulation (*Bhlhe40* KO: 85 ± 12.8%, WT: 68.5 ± 19.5%; p<0.001; n = 5 mice, 6 slices each). Data from Figs 3A, 3B, 3C, and 3D were p<0.05 on Shapiro Wilk normality test and tested for significance by Mann-Whitney Rank Sum Test. Error bars are standard error of the mean.

In field recordings from hippocampal area CA1, input/output curves revealed a greater postsynaptic response (EPSP slope) to axon input stimulation (fiber volley amplitude) in WT mice compared to *Bhlhe40* KO mice indicating a greater intrinsic response to stimulation (Fig 3A). There was no difference between WT and *Bhlhe40* KO mice in paired pulse facilitation (Fig 3B) indicating no difference in glutamate release from presynaptic terminals. There was a significant reduction in both LTP (60% decrease; p<0.001; Fig 3C) after high frequency stimulation (100 Hz, 1 s) and LTD (52% decrease; p<0.001; Fig 3D) after low frequency stimulation (1 Hz, 15 min) in *Bhlhe40* KO hippocampal slices compared to controls. Combined, these findings indicate a decrease in long-term synaptic plasticity due to post-synaptic mechanisms.

**Fig 4:**
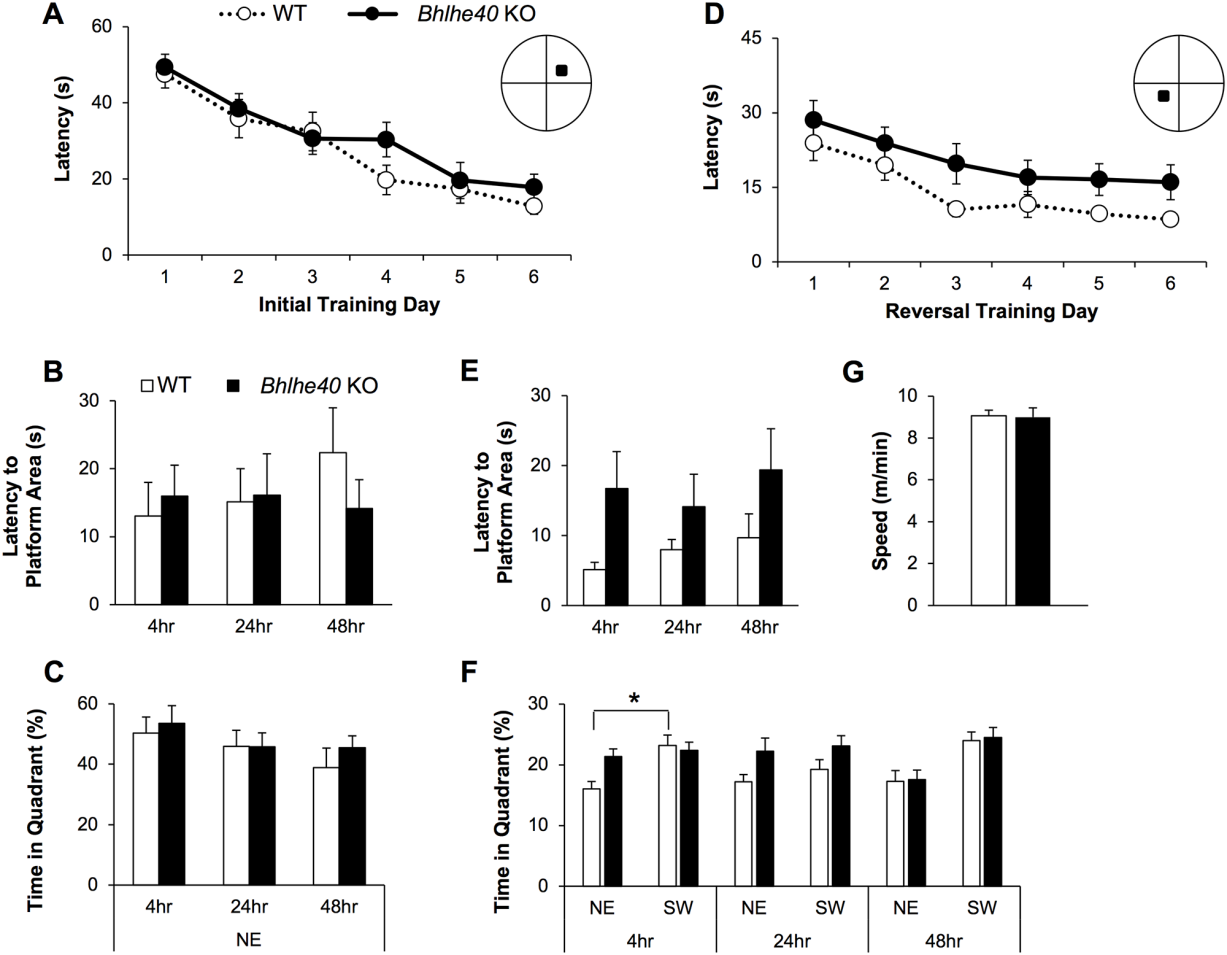
*Bhlhe40* KO mice have impaired relearning on the MWM. (A) There was no change in escape latency during initial learning and (B) initial probes revealed no cognitive deficits of *Bhlhe40* KO animals as indicated by latency to platform. (C)or time spent in the goal (NE) quadrant. (D) *Bhlhe40* KO mice had impaired relearning of the new platform location (SW quadrant) (linear regression line elevation difference p<0.01) (two-way RM-ANOVA p = 0.067). (E) Similarly, *Bhlhe40* KO animals were impaired in the latency to the new platform area on the reversal probes (linear regression line elevation difference p<0.05 t-test) (two-way RM-ANVOA p = 0.059). (F) On the 4hr reversal probe *Bhlhe40* KO showed no preference for the new platform location (SW) quadrant compared to the initial platform location (NE) quadrant, whereas WT animals spent more time in the NE compared to SW quadrants (p<0.05). (G) There was no significant difference in time spent in the SW quadrant compared to the NE quadrant for either *Bhlhe40* KO or WT animals on the 24hr and 48hr reversal probes (p>0.05), although there was a near-significant trend for WT animals on the 48hr probe (p = 0.0531). Across all conditions there was no effect on swim speed (p>0.05) WT n = 12, *Bhlhe40* KO n = 12. Initial 24hr probe measurements included only n = 11 for *Bhlhe40* KO mice due to a software error in recording one animal. One *Bhlhe40* KO animal was removed from the dataset due to repeated thigmotaxis-like behavior. Error bars are standard error of the mean. Statistical significance (*p<0.05) was tested by two-way RM-ANOVA (Figs 4A, 4B, 4C, 4D, 4E, and 4F), comparison of linear regression lines (Figs 4A, 4B, 4D, and 4E) and Mann Whitney U test (Figs 4E, 4F, and 4G).

Impairments in synaptic plasticity are often thought to often underlie problems in learning and memory [28], therefore we next investigated whether *Bhlhe40* KO mice, which have reductions in hippocampal synaptic plasticity (Fig 3), also have impairments in learning and memory on the Morris Water Maze (MWM), a hippocampal-dependent spatial navigation task. We trained *Bhlhe40* KO and WT mice to find the hidden platform, located in the NE quadrant, in the MWM for 6 days (Initial Training), then administered probe tests at 4-, 24-, and 48hrs, where the animals were allowed to search for the platform for 60 seconds without an escape platform (Initial Probes). We found no significant difference between genotype groups on either the latency to escape from the water during initial training (Fig 4A) or on the initial probes (Fig 4B and 4C). Next, 14 days from the start of the MWM experiment, we trained animals to find the platform located in the opposite (SW) quadrant of the MWM for another 6 days (Reversal Training) (Fig 4D), followed by another series of probe tests at 4-, 24-, and 48hrs (Reversal Probes) (Fig 4E and 4F). We found a non-significant trend in re-learning impairment on the reversal learning curve (p = 0.067) by two-way ANOVA (genotype x reversal training days), and a significant difference in the elevation between the linear regression lines of *Bhlhe40* KO and WT animals (p<0.01) (Fig 4D, regression lines not shown). On the reversal probes there was a non-significant trend overall on the latency to where the platform had been in the SW quadrant across probe trials (p = 0.059 by two-way RM-ANOVA; Fig 4E), as well as a significant difference in the elevation of the linear regression lines between *Bhlhe40* KO and WT (p<0.05; Fig 4E, regression lines not shown). In addition, we found a significant difference in the elevation of the *Bhlhe40* KO and WT linear regression lines for the latency to the NE quadrant, where the animals had been trained to find the platform on the initial training (p<0.05, data not shown). This persistent tendency toward the initial platform location in *Bhlhe40* KO animals is also reflected in the 4hr reversal probes, where *Bhlhe40* KO showed no preference for the new platform (SW) location compared to the initial platform (NE) location, measured by percent time in quadrant, whereas WT mice spent more time in the new goal (SW) quadrant (Fig 4F, p<0.05). A similar trend for WT animals was also observed on the 48hr probe (p = 0.0531). There was no effect on platform crossings (data not shown) or swim speed (Fig 4G). Together, these data suggest that *Bhlhe40* KO animals have an impairment in relearning.

### Hippocampi of *Bhlhe40* KO mice have altered basal gene expression profiles for energy metabolism, insulin regulation, and neuronal signaling

**Fig 5:**
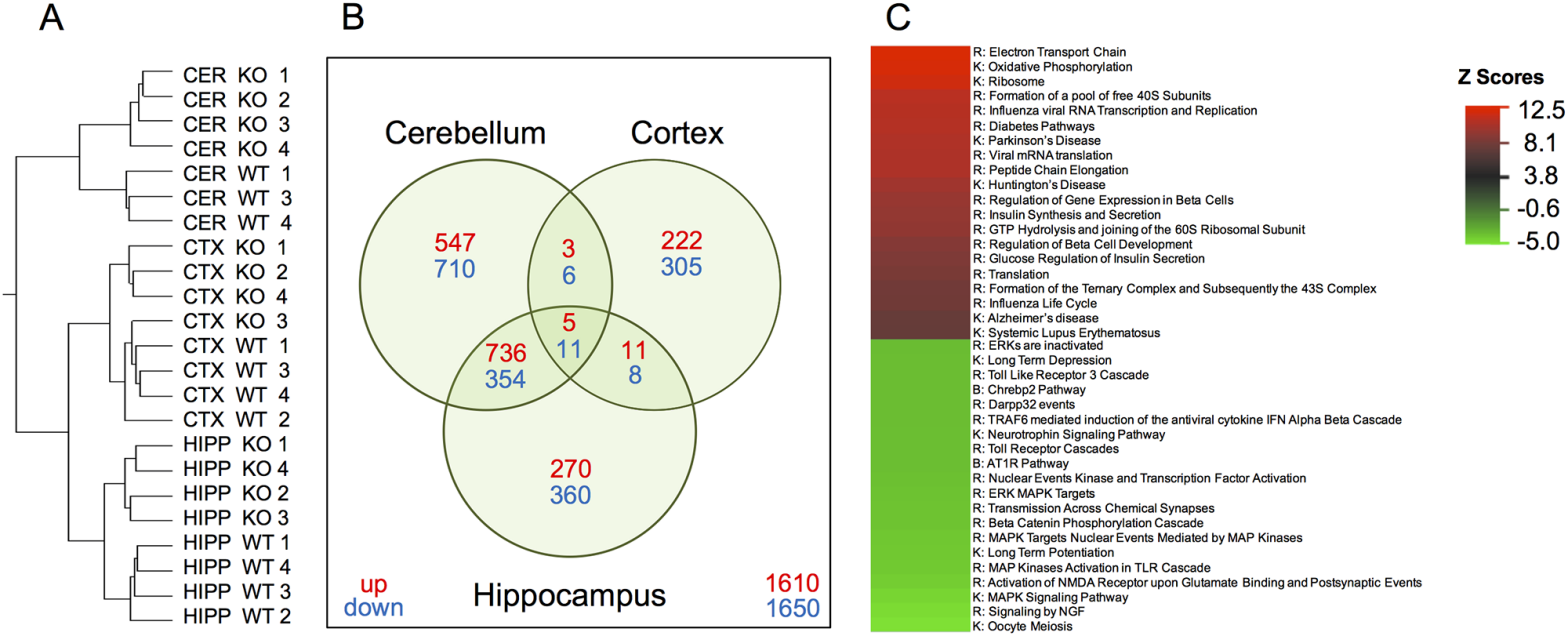
*Bhlhe40* KO mice have unique changes in gene expression in the central nervous system. Unilateral Hippocampus (HIPP), Cortex (CTX), and Cerebellum (CER) were isolated from naïve *Bhlhe40* KO and WT animals (n = 4). MRNA was extracted and run on an Illumina whole genome expression array. (A) *Bhlhe40* KO and WT hippocampal gene expression had non-overlapping separation. One WT CER sample was removed as an outlier and one *Bhlhe40* KO CTX clustered with the WT samples but was included in the analysis. (B) Venn Diagram of significant genes changed (p<0.05, FDR<0.3) in *Bhlhe40* KO Cerebellum, Cortex, and Hippocampus, relative to WT). (C) Pathway analysis of a whole genome expression array on hippocampal genes identified several highly upregulated pathways in energy metabolism and downregulated pathways involved in synaptic activity in *Bhlhe40* KO mice compared to WT mice. The top 20 upregulated and top 20 downregulated pathways are shown, organized by Z score; n = 4. R = Reactome; K = Kegg; B = Biocarta.

Next, we wanted to investigate in what ways Bhlhe40 gene deletion may be leading to increased neuronal excitability and reduced synaptic plasticity in hippocampal slices. To address this, we performed a whole genome expression array of the hippocampus, as well as cortex and cerebellum from naïve 4-month old WT and *Bhlhe40* KO mice (n = 4 each). Although we expected minimal changes in the cerebellum due to its lower levels of *Bhlhe40* gene expression compared to hippocampus and cortex [9], we were surprised to find that all three tissues had a high number of statistically significant differences in mRNA levels (Fig 5B). Notably, changes in gene expression from *Bhlhe40* gene deletion are unique in the hippocampus and hierarchical clustering revealed a clean separation pattern between *Bhlhe40* KO and WT hippocampal RNA gene expression (Fig 5A).

Focusing on hippocampal gene expression, an in-depth analysis showed an enrichment in pathways involved in energy metabolism and insulin signaling amongst the top 20 upregulated pathways in *Bhlhe40* KO hippocampus (e.g. Reactome electron transport chain, Reactome diabetes, Reactome insulin synthesis and secretion, and Reactome glucose regulation of insulin secretion; Fig 5C). In addition, several pathways pertaining to neuronal signaling were included in the top 20 downregulated pathways (e.g. Kegg long term potentiation, Kegg long term depression, Reactome transmission across chemical synapses, and Reactome activation of NMDA receptor upon glutamate binding and postsynaptic events; Fig 5B).

### Quantitative RT-PCR and ChIP-Seq analysis of *Bhlhe40* KO and WT hippocampus reveals differential patterns of expression consistent with direct repression/de-repression of circadian genes and indirect regulation of insulin degrading enzyme (*Ide*) gene expression

We then used the whole genome expression array data for hippocampus to search for gene expression changes that may underlie the neuronal excitability and synaptic plasticity results we obtained in hippocampal slices (Figs 1 and 3). Of the genes predicted to be significantly changed in *Bhlhe40* KO hippocampi by whole genome expression array, we chose a subset of genes to validate based on their predicted fold-change, level of significance, and their known role in neuronal excitability, seizure activity, or neurological dysfunction. We used qRT-PCR to validate a number of genes whose mRNA levels were significantly changed based on the gene expression microarray screen. For this validation, we used contralateral hippocampus from the same four *Bhlhe40* and WT animals for the gene expression array plus another four samples (n = 8; 3–5 months old). We were interested in genes encoding membrane receptors or other proteins that may be involved in neuronal excitability or synaptic plasticity: Glutamate receptor 1 (*Gria1*) [29], GABA(B) receptor 1 (*Gabbr1*) [30], the potassium channel genes *Kcnh1* [31], *Kcnh2* [32], *Kcnab1* [33], the sodium channel subunit 1a (*Scn1a*) [34], the neurotrophic receptor tyrosine kinase 3 (*NTrk3*), which is involved in glutamatergic synapse formation [35], the protein kinase calmodulin kinase 1d (*Camk1d*), which activates cyclic AMP Response Element Binding Protein (CREB) in hippocampal neurons [36], the cell adhesion molecule *Chl1* involved in synaptic vesicle recycling [37], and PGC-1alpha (*Ppargc1a*), which is involved in mitochondrial biogenesis in neurons [38], and insulin degrading enzyme (IDE), which degrades peptides such as insulin and is involved in peptide signaling and is a modulator of synaptic plasticity [39]. We additionally wanted to examine transcripts for the circadian regulators *Clock* [40], *Dbp* [41] and *Bhlhe41*, the sister protein of *Bhlhe40* [7], as Bhlhe40 is well established as a circadian regulator [7] and circadian dysregulation may cause hyperexcitability [42] as well as alterations in synaptic plasticity [43].

**Fig 6:**
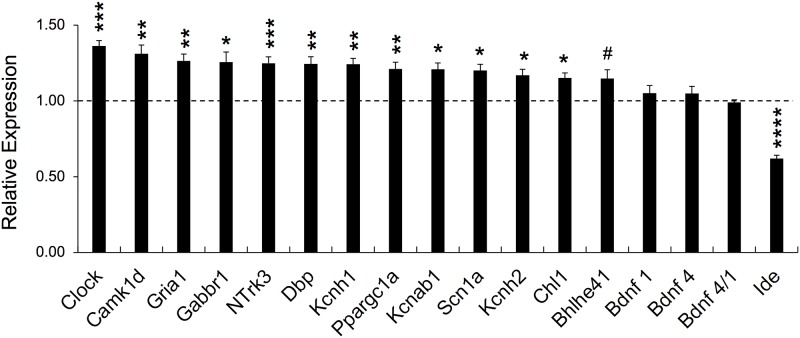
*Ide* is downregulated in *Bhlhe40* KO hippocampus. For validation, messenger RNA (mRNA) levels were determined by Quantitative Reverse Transcription Polymerase Chain Reaction (qRT-PCR). Messenger RNA from WT (n = 8) and *Bhlhe40* KO (n = 8) mice were extracted from hippocampal tissue. Messenger RNA levels for genes in *Bhlhe40* KO mice are expressed relative to WT (100% = dashed line). Specific mRNA levels within samples were normalized to the housekeeping gene *Hprt*. *Bdnf 4* was additionally tested relative to *Bdnf 1* (*Bdnf 4/1*). Unpaired t-tests were used to test for significance for each gene; * = p<0.05, **p<0.01, *** = p<0.001, **** = p<0.0001 (S1 Table for all genes tested); WT n = 8, *Bhlhe40* KO n = 8; Error bars are standard error of the mean. All error bars are standard error of the mean.

We also investigated *Bdnf* transcripts 1 and 4, because of the previously published interaction between Bhlhe40 protein and promoter 4 of the *Bdnf* gene [11]. By qRT-PCR analysis we found significantly upregulated transcripts in *Clock* (p<0.001) *Camk1d* (p<0.01), *Gria1* (p<0.01), *Gabbr1* (p<0.05), *NTrk3* (p<0.001), *Dbp* (p<0.01), *Kcnh1* (p<0.01), *Kcnh2* (p<0.05), *Ppargc1a* (p<0.01), *Scn1a* (p<0.05), *Kcnab1* (p<0.05), and *Chl1* (p<0.05) in *Bhlhe40* KO hippocampi (Fig 6). In addition, *Bhlhe41* mRNA levels trended towards an increase that was not quite significant (15% upregulated p = 0.06) (Fig 6). We found no significant difference in hippocampal *Bdnf* transcripts 1 or 4 by qRT-PCR (Fig 6). There was also no change in *Bdnf4* relative to *Bdnf1* levels (*Bdnf* 4/1) (Fig 6). *Ide* was 1.6-fold downregulated (p<0.0001) (Fig 6). *Ide* was the only gene we found to have significantly downregulated expression and was the most significantly changed of all 33 genes tested by qRT-PCR (see S1 Table) for a list of all genes tested by qRT-PCR and their corresponding gene expression array values.

To approach potential mechanisms for describing the qRT-PCR findings in the *Bhlhe40* KO mice on the C57Bl/6 genetic background, we performed a genome-wide chromatin immunoprecipitation sequence (ChIP-Seq) analysis using hippocampi from 2.5 month old male WT mice. Due to limitations in the amount of tissue available for this experiment, we pooled hippocampi from the WT males (all were of the same age) for Bhlhe40 antibody ChIP and for input controls. Bhlhe40 occupancy data for the differentially expressed genes validated by qRT-PCR are summarized in S2 Table. Since Bhlhe40 is a known regulator of circadian function [7], it was predicted that regulatory regions for the *Clock* and *Dbp* genes would show Bhlhe40 occupancy. As predicted, there were increased levels of Bhlhe40 bound to the 5’ flanking region of these two genes above input control with peak values of 22 for *Clock* and 73 for *Dbp* at CACGTG E-box containing sites. Intriguingly, Bhlhe40 was bound to DNA within the *Bdnf* gene (peak value 20 above input control), but at a different E-box site than expected (within intron 3; S2 Table), distal from the 5’ flanking region regulatory site seen in mixed background mice [11]. Another gene evaluated in the whole genome expression array screen, the *Bhlhe40* family member, *Bhlhe41*, also showed significant levels of Bhlhe40 occupying its respective 5’ regulatory region (two sites, average peak value 41.4 above input control). This result suggests a direct effect on transcription, like the *Clock* and *Dbp* genes. Other genes showing significant differences between WT and *Bhlhe40* KO from the expression array screen, and validated by qRT-PCR, namely *Camk1d*, *Gabbr1*, *Gria1*, *Kcnh1* and *Kcnab1* had Bhlhe40 bound to DNA but within the gene body or occupying the 3’ flanking region. Lastly, there were qRT-PCR validated genes with no evidence of Bhlhe40 occupancy across any portion of the gene: *Chl1*, *Ide*, *Kcnh2*, *NTrk3*, *Ppargc1a*, *and Scn1a*. Taken together, these findings support the idea that enhanced levels of *Clock*, *Dpb*, and *Bhlhe41*-specific mRNAs in *Bhlhe*40 KO mice may be the result of direct transcriptional de-repression. In contrast, genes like *Ide*, which had the most significantly changed level of mRNA of any gene described in this study, did not have significant levels of Bhlhe40 associated with any part of its gene sequence. This finding suggests that Bhlhe40 likely regulates *Chl1*, *Ide*, *Kcnh2*, *NTrk3*, *Ppargc1a*, *and Scn1a* transcription indirectly. A list of all genes and gene intervals occupied by Bhlhe40 protein in WT hippocampus is shown in S3 Table.

### Insulin degrading enzyme protein is decreased in *Bhlhe40* KO hippocampus

**Fig 7:**
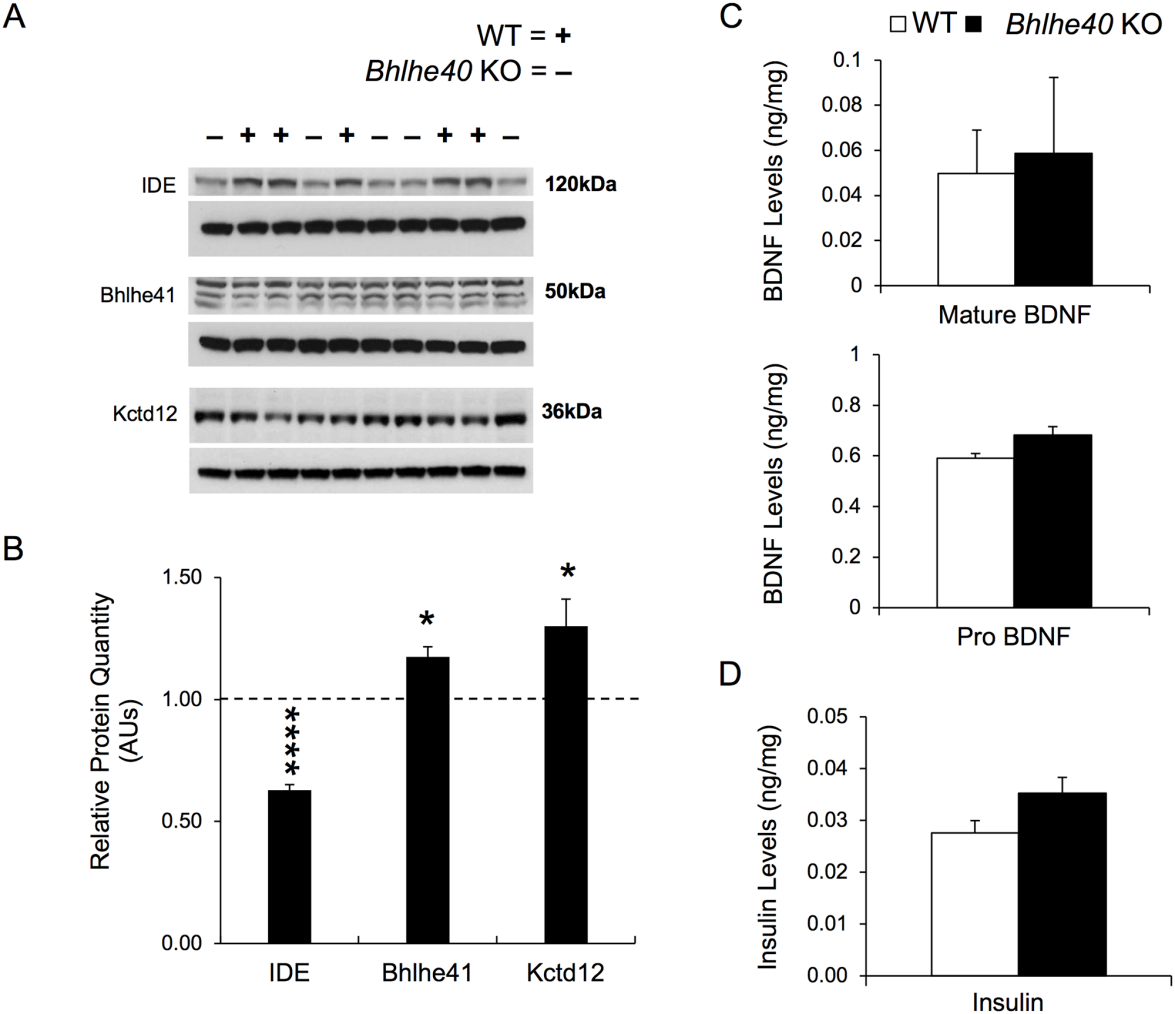
Insulin levels trended to an increase but are not changed in the hippocampus of *Bhlhe40* KO mice. (A) Protein (Western) Blots were used to determine expression levels of proteins for candidate genes identified from the mRNA validation experiments. Single hippocampi from six WT and *Bhlhe40* KO mice were used for protein-level analysis by western blotting. Representative autoradiograms from WT (+) and *Bhlhe40* KO (-) samples are shown for IDE (120kDa), Bhlhe41(50kDa), and Kctd12 (36kDa). (B) Protein quantification by western blot was determined via densitometry using Fiji (Image J). In *Bhlhe40* KO hippocampus, IDE was 1.6-fold downregulated (p<0.0001), Kctd12 was 30% upregulated (p<0.05), and Bhlhe41 was 18% upregulated (p<0.05) relative to WT levels. For Bhlhe41, a triplet band was observed, likely due to the two possible post translation sumoylations. The relative expression for Bhlhe41 represents a combination of all three bands. Unpaired t-tests were used to test for significance changes for protein levels. Values shown are *Bhlhe40* KO protein level relative to WT levels by arbitrary units (AUs); * = p<0.05 **** = p<0.0001. Additionally, Clock, Camk1d, Gabbr1, GluR1, Scn1a, and Chl1 were tested and found to be not significantly changed in *Bhlhe40* KO hippocampus. (C) Single hippocampi from six WT and *Bhlhe40* KO mice were used for BDNF quantification by ELISA. Mature BDNF (top) and Pro BDNF (bottom) were tested on independent ELISAs. BDNF levels are expressed in ng of BDNF relative to mg of total protein. Top) pro BDNF (unpaired t-test p>0.05); Bottom) mature BDNF (unpaired t-test p>0.05). (D) Single hippocampi from seven WT and *Bhlhe40* KO mice were used for insulin quantification by ELISA. Insulin levels are expressed in ng of insulin relative to mg of total protein (insulin ELISA students t-test p = 0.07). All error bars are standard error of the mean.

We next investigated whether mRNA-level changes in *Bhlhe40* KO hippocampal gene expression translated to protein level changes in hippocampus. 7 of the 13 genes found to have significantly different expression on the RNA level (Fig 6) were tested for protein level changes by western blot, i.e. IDE, Clock, Camk1d, Gabbr1, GluR1 (*Gria1*), Scn1a, and Chl1. Of those seven proteins, only IDE was found to be significantly altered with a highly significant 1.6-fold decrease in *Bhlhe40* KO hippocampal IDE protein levels relative to WT hippocampi (p<0.0001) (Fig 7A and 7B). We also found that Bhlhe41, which had only a non-significant increase on the RNA level (15% upregulated p = 0.06 by qRT-PCR; Fig 6), was upregulated by 18% in *Bhlhe40* KO hippocampus (p<0.05) (Fig 7A and 7B). Bhlhe41 protein blotting generated three bands, consistent with the two known post-translational sumoylations [6], which were combined for densitometry analysis to determine the total fold change. Additionally, Kctd12 was tested for protein level differences in *Bhlhe40* KO hippocampi by western blotting and was found to have a 30% increase in protein levels relative to WT (p<0.05) (Fig 7A and 7B). Whole genome expression array of *Bhlhe40* KO hippocampal RNA predicted Kctd12 to be differentially expressed, however, because Kctd12 contains only a single exon, it was not tested for changes on the RNA level by qRT-PCR, as we only used intron-spanning primers. There were no significant differences in Clock, Camk1d, Gabbr1, or GluR1, Scn1a, or Chl1 (see S1 Table for the full all proteins tested and their corresponding gene expression array and qRT-PCR values). GAPDH was used as a reference control for all proteins tested and was consistent between WT and *Bhlhe40* KO protein samples.

In light of our previous observation that *Bdnf* mRNA and protein levels are elevated in *Bhlhe40* KO mice on a mixed genetic background [11], we sought to determine if there was a difference in protein levels of mature BDNF or proBDNF in *Bhlhe40* KO hippocampus at baseline. Protein extracts from six WT and six *Bhlhe40* KO hippocampi were assayed on independent ELISAs for mature BDNF and proBDNF. There was no significant difference in protein level between the genotype groups for either form of BDNF between *Bhlhe40* KO and WT hippocampi (Fig 7C).

Given our findings of decreased IDE protein levels in *Bhlhe40* KO hippocampus, we wanted to determine if there was an increase in IDE substrate levels, namely insulin, in *Bhlhe40* KO mice via an insulin ELISA. We used seven WT and seven *Bhlhe40* KO hippocampal samples. *Bhlhe40* KO hippocampal insulin levels trended toward an increase (p = 0.07), relative to WT hippocampal insulin levels, but were not significant (Fig 7D).

## Discussion

We found that *Bhlhe40* KO mice exhibited increased excitability in CA1 neurons but observed no change in seizure severity from intra-hippocampal KA injection. This suggests that *Bhlhe40* gene deletion leads to enhanced neuronal excitability in at least one component of the hippocampal circuit. However, this increased excitability was not sufficient to increase vulnerability to seizures originating in the hippocampus. This finding is partially contrasted by our earlier report that found that *Bhlhe40* KO-mix mice have enhanced seizure responses when injected intraperitoneally with KA [11]. It is plausible that differences in delivery routes, intraperitoneal injection versus intrahippocampal injection, could explain differences in response in the KA model of temporal lobe epilepsy. However, due to the density of the KA receptors, GluK4 and GluK5 in CA1 and CA3 regions of the hippocampus, the hippocampus is typically the region of seizure generation regardless of the route of injection [10]. Given that *Bhlhe40* is strongly expressed in CA1 of the hippocampus [9], it is most likely that differential seizure responses between the *Bhlhe40* KO on a congenic C57Bl/6 background (reported here) and the *Bhlhe40* KO on a mixed genetic background (129, C57Bl/6, and CD1; [11]) models are due to changes in the genetic background. While use of congenic mice is important for standardization of animal models, it should be noted that C57Bl/6 mice are more resistant to seizure induction than some other genetic backgrounds [44]. Together, this suggests that changes to the genetic background of the *Bhlhe40* KO mice is likely responsible for loss of enhanced seizure vulnerability. Importantly, Jiang et al., proposed that de-repression of the *Bdnf* gene at promoter 4 in the hippocampus was a possible mechanism for the increased seizure susceptibility observed in *Bhlhe40* KO mice on a mixed genetic background [11]. Here, using congenic C57Bl/6 *Bhlhe40* KO mice we found no change in *Bdnf 1* or *Bdnf 4* gene expression, or BDNF protein levels; however, by ChIP-Seq we found Bhhe40 binding to the *Bdnf* gene, but at a more distant site than observed in the mixed background *Bhlhe40* KO mice [11].

At the nucleotide level, variation in the sequence of promoter regions or sequences between promoter elements in Bhlhe40 target genes could have a direct effect on transcription. Strain-specific binding patterns for transcription factors can be determined empirically by ChIP-Seq. These specific changes can also act at long range [45]. For example, one mechanism involving Bhlhe40-mediated transcriptional repression acts on the *p53* gene by direct interaction, but only if the *Bhlhe40* and *p53* promoter elements are within a few base pairs of each other [46]. Therefore, it is likely that inter-strain genetic variation could influence promoter spacing and thus Bhlhe40 interactions. This could account for the differences in *Bdnf* gene promoter occupancy and *Bdnf* mRNA levels we observed between *Bhlhe40* KO and *Bhlhe40* KO-mix.

In hippocampal slices from *Bhlhe40* KO mice, we observed a decrease in synaptic plasticity at CA1 synapses and saw an impairment in relearning on the MWM, although no difference on initial training. The lack of difference in performance between *Bhlhe40* KO mice and controls on the MWM initial training is not surprising and corroborates what has been seen previously in *Bhlhe40/Bhlhe41* KO mice [47]. NMDARs are key players in hippocampal synaptic plasticity [48]. Conditional deletion of GluN1, an obligatory subunit of NMDARs, in the dentate gyrus and CA1-3 principal neurons (*GluN1*^Δ*DGCA1*^), resulted in significantly reduced LTP, but not behavioral deficits on MWM initial training [49]. *GluN1*^Δ*DGCA1*^ mice, however, did exhibit deficits on the MWM reversal task [49], similar to what we have shown here in *Bhlhe40* KO mice.

In gene expression array validation experiments, IDE stood out as highly significantly decreased in *Bhlhe40* KO hippocampus. In our ChIP-Seq experiment we did not find Bhlhe40 bound to the *Ide* gene *in vivo*, suggesting Bhlhe40 indirectly regulates the expression of *Ide*. Despite the 1.6-fold decrease in IDE protein levels, however, there was no significant increase in hippocampal insulin levels in *Bhlhe40* KO mice, suggesting that 63% of WT IDE protein levels is sufficient to maintain normal insulin homeostasis in 4–7 month old mice. IDE, however, is known to have other substrates, including: Igf-1 and Igf-2, amylin, and Amyloid ß [50]. Notably, it was previously found that *Bhlhe40/Bhlhe41* KO mice have enhanced Igf-2 signaling in cortex [47]. Reductions in IDE levels may underlie the enhanced Igf-2 signaling seen in *Bhlhe40/Bhlhe41* KO mice.

In summary, we propose a novel role for Bhlhe40 in hippocampal synaptic plasticity and neuronal excitability based on a balance of direct and indirect transcriptional effects. However, attempts to generalize our findings are complicated by strain-associated differences. Future studies should associate genetic variants between mouse strains with gene expression in different brain regions in order to better understand how Bhlhe40 affects synaptic plasticity and neuronal excitability.

## Supporting information

S1 Fig *Bhlhe40* KO CA1 neurons have no difference in mEPSC frequency. Whole-cell patch clamp recordings of CA1 neurons from *Bhlhe40* KO hippocampal slices revealed no significant difference in mEPSC frequency; n = 4 mice for each *Bhlhe40* KO (5 cells) and WT (6 cells). Error bars are standard error of the mean; unpaired t-test p>0.05.
(TIF)

S2 Fig *Bhlhe40* KO mice do not have more neuronal cell death following KA infusion. Neuronal death was quantified using Fiji software to measure integrated density of staining of the CA3 pyramidal neuron cell body layer relative to background staining (A; n = 4; unpaired t-test p>0.05). Thickness of the CA3 cell body was assessed using Fiji software by measuring the length across the CA3 cell body layer to measure neuronal apoptotic swelling (B; n = 4; unpaired t-test p>0.05).
(TIF)

S3 Fig EEG responses to KA and saline infusion. (A) Example Traces of EEG activity six hours following saline and KA infusion in WT and *Bhlhe40* KO animals. The same animals are displayed for the saline and KA delivery for the WT and *Bhlhe40* KO examples. The Y axis for the EEG figures are +2mV to -2mV for each. EEG power over time for the first 6 hours at 5-minute intensity averages after (B) saline, WT (n = 6) and *Bhlhe40* KO (n = 7) and (C) KA, WT (n = 6) and *Bhlhe40* KO (n = 5; 2 outliers pulled). (D) 15-minute intensity averages for 24 hours following KA infusion. (Fig 7B, 7C and 7D) 1–4 Hz activity is a surrogate for seizure scores 1–3, and 6-10Hz activity is a surrogate for seizure scores 4–6. 30-50Hz activity is known to precede seizure activity and is an additional ictal marker. Repeated measures two-way ANOVA was used for each dataset corresponding to Fig 7B, 7C and 7D; p>0.05 for all. Error bars are standard error of the mean.
(TIF)

S1 Table Differentially expressed genes in *Bhlhe40* KO hippocampi relative to WT hippocampi. This table shows gene-expression changes in *Bhlhe40* KO hippocampi relative to WT hippocampi across conditions, from the gene expression array to the qRT-PCR conditions and protein quantification where applicable. All fold changes and p values are shown. For qRT-PCRs p-values represent unpaired t-tests for gene expression relative to the reference gene expression from *Hprt*. Gene expression array data also indicates the false detection rate (FDR) for each gene.
(XLSX)

S2 Table ChIP-Seq analysis. This table shows the ChIP-Seq data for genes of interest from the qRT-PCR experiment.
(XLSX)

S3 Table All Bhlhe40 ChIP-Seq data. List of all genes and gene intervals occupied by Bhlhe40 in WT hippocampus by ChIP-Seq.
(XLSX)
