# Supplementary figures and images for: Mice lacking the transcriptional regulator Bhlhe40 have enhanced neuronal excitability and impaired synaptic plasticity in the hippocampus
Source: PLoS One. 2018 May 1;13(5):e0196223. doi: 10.1371/journal.pone.0196223 (PMC5929507; doi:10.1371/journal.pone.0196223)

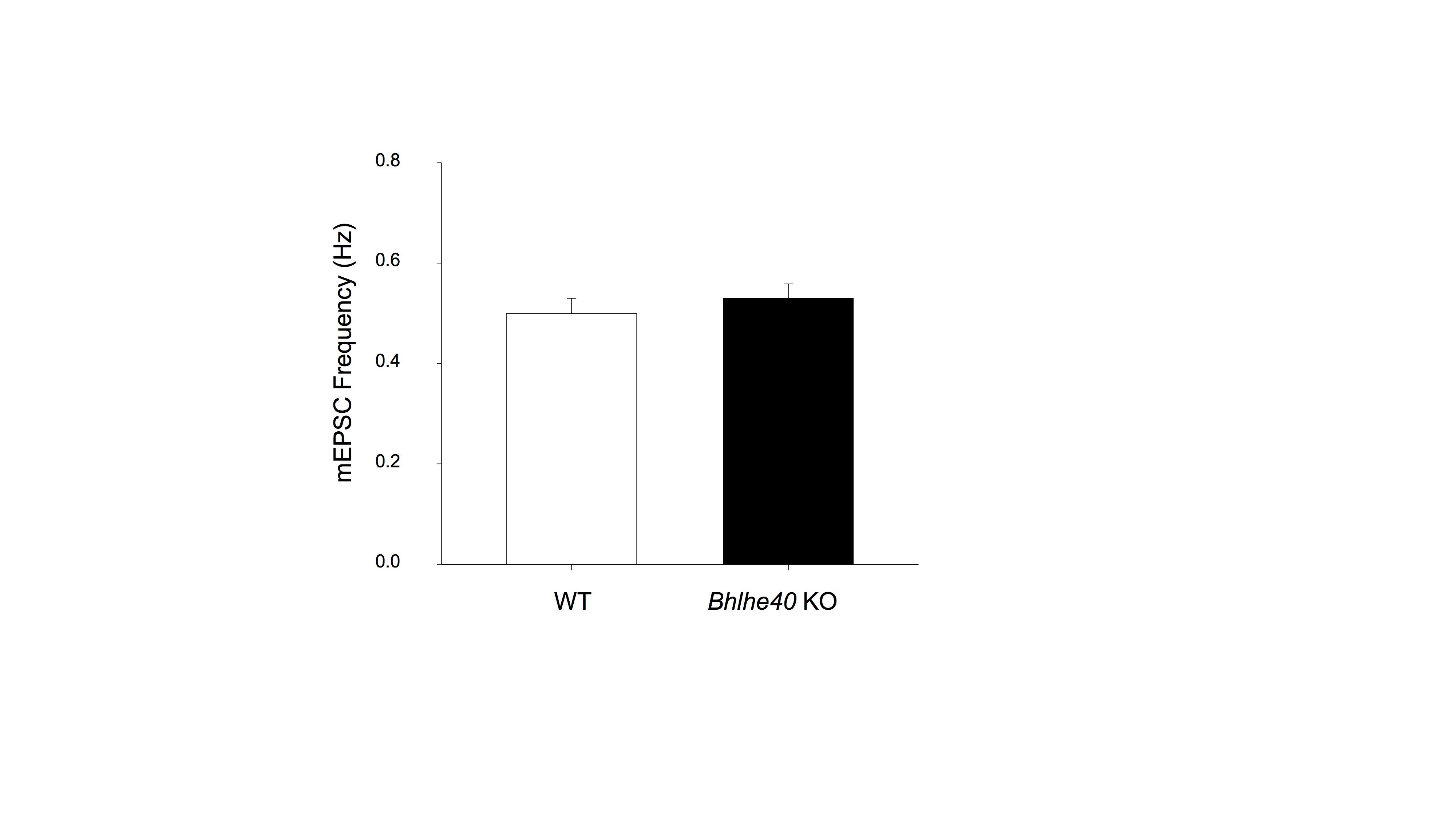

Supplement: S1 Fig — Whole-cell patch clamp recordings of CA1 neurons from Bhlhe40 KO hippocampal slices revealed no significant difference in mEPSC frequency; n = 4 mice for each Bhlhe40 KO (5 cells) and WT (6 cells). Error bars are standard error of the mean; unpaired t-test p>0.05. (TIF) [file pone.0196223.s001.tif]

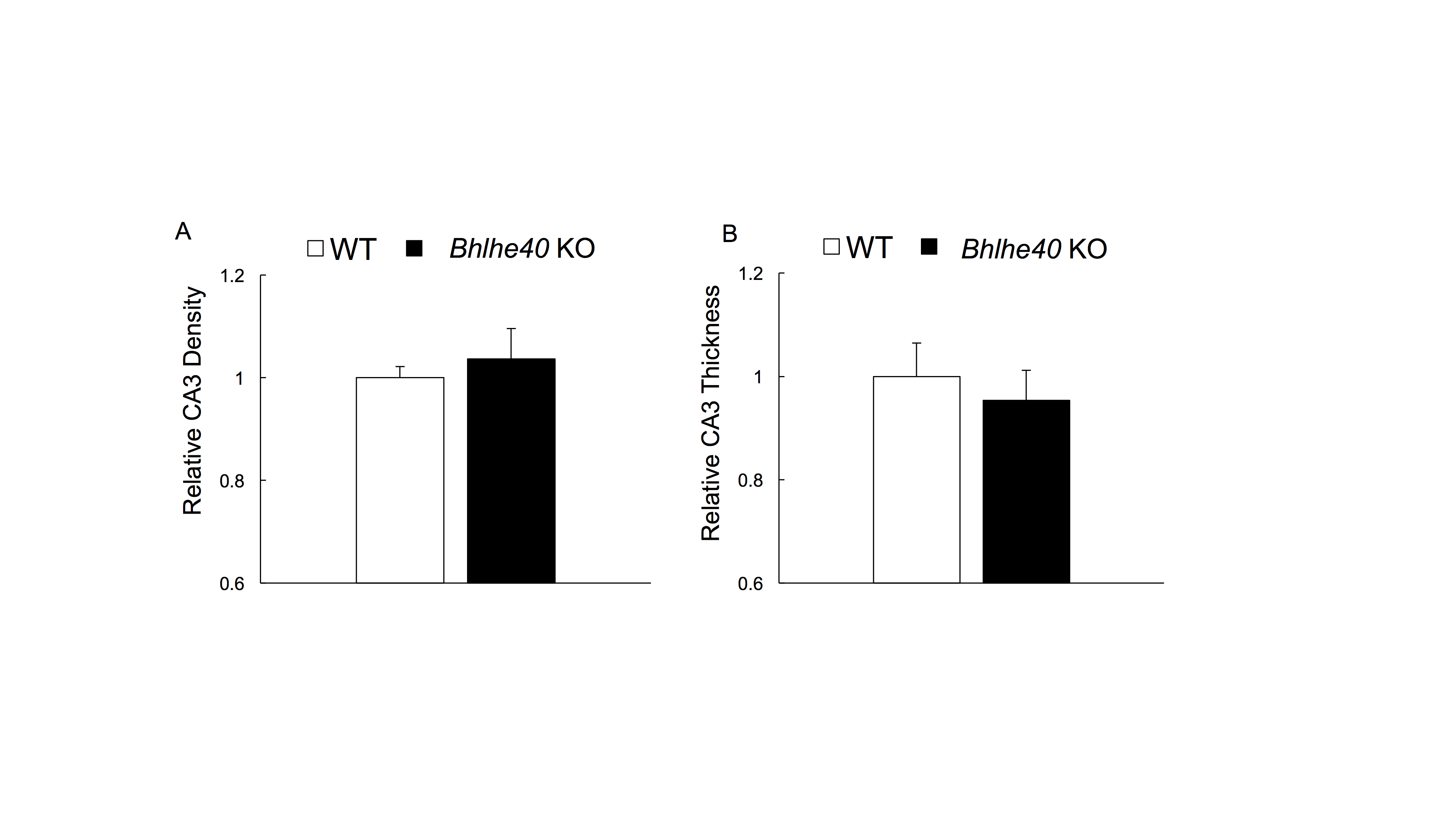

Supplement: S2 Fig — Neuronal death was quantified using Fiji software to measure integrated density of staining of the CA3 pyramidal neuron cell body layer relative to background staining (A; n = 4; unpaired t-test p>0.05). Thickness of the CA3 cell body was assessed using Fiji software by measuring the length across the CA3 cell body layer to measure neuronal apoptotic swelling (B; n = 4; unpaired t-test p>0.05). (TIF) [file pone.0196223.s002.tif]

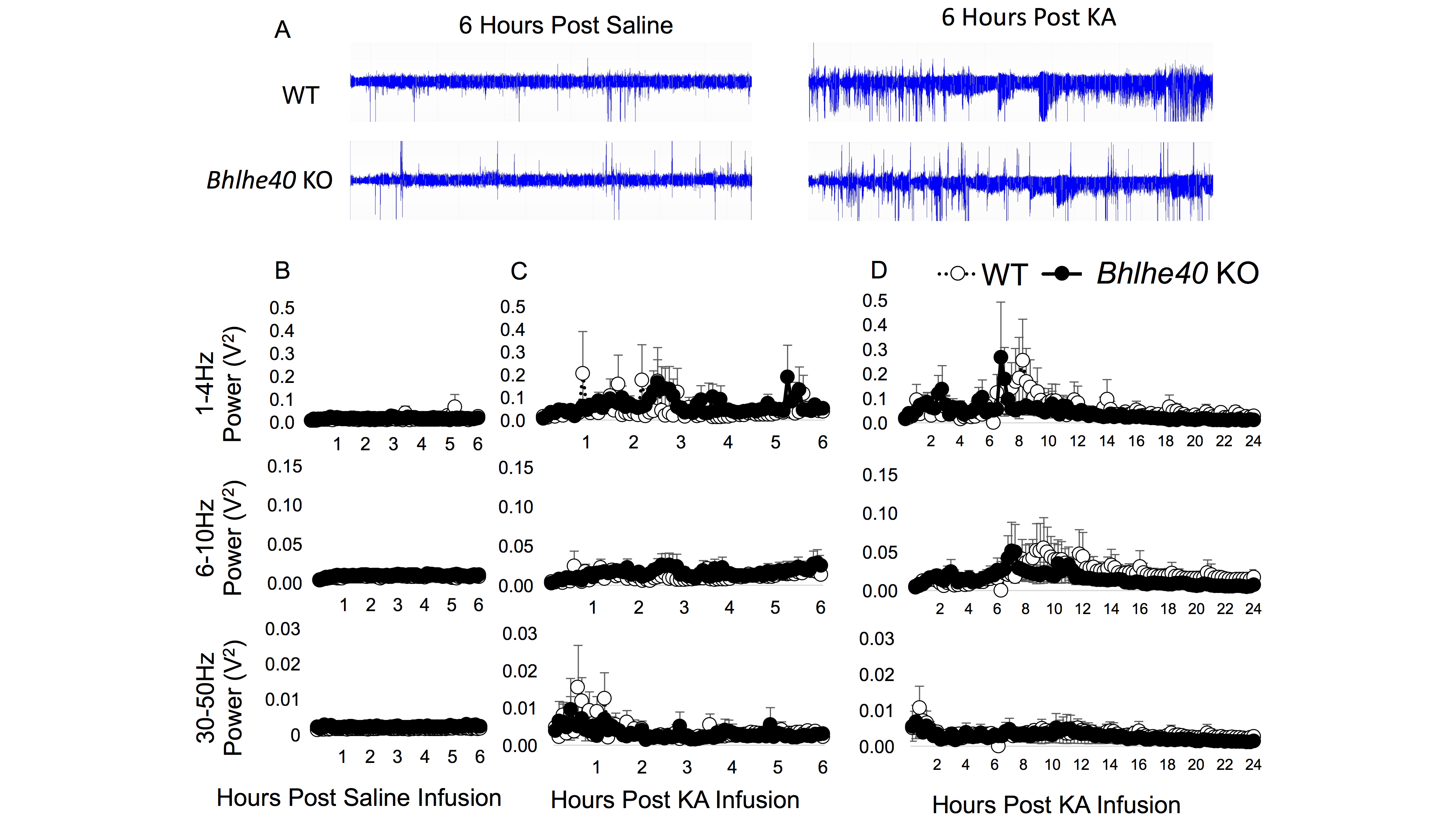

Supplement: S3 Fig — (A) Example Traces of EEG activity six hours following saline and KA infusion in WT and Bhlhe40 KO animals. The same animals are displayed for the saline and KA delivery for the WT and Bhlhe40 KO examples. The Y axis for the EEG figures are +2mV to -2mV for each. EEG power over time for the first 6 hours at 5-minute intensity averages after (B) saline, WT (n = 6) and Bhlhe40 KO (n = 7) and (C) KA, WT (n = 6) and Bhlhe40 KO (n = 5; 2 outliers pulled). (D) 15-minute intensity averages for 24 hours following KA infusion. (Fig 7B, 7C and 7D) 1–4 Hz activity is a surrogate for seizure scores 1–3, and 6-10Hz activity is a surrogate for seizure scores 4–6. 30-50Hz activity is known to precede seizure activity and is an additional ictal marker. Repeated measures two-way ANOVA was used for each dataset corresponding to Fig 7B, 7C and 7D; p>0.05 for all. Error bars are standard error of the mean. (TIF) [file pone.0196223.s003.tif]
